# The phenine concept delivers a nitrogen-doped nanotube and evokes infinite possibilities

**DOI:** 10.1038/s42004-020-00348-3

**Published:** 2020-07-28

**Authors:** Joshua C. Walsh, Graham J. Bodwell

**Affiliations:** Chemistry Department, Memorial University of Newfoundland, St. John’s, NL Canada A1B 3X7

**Keywords:** Carbon nanotubes and fullerenes, Synthesis and processing, Structural properties

## Abstract

Isobe et al. recently introduced the phenine concept, whereby a 1,3,5-trisubstituted benzene system (a phenine unit) is used as a basic building block instead of an *sp*^2^-hybridized carbon atom. Now, they apply it in a concise synthesis of a nitrogen-doped phenine nanotube.

Before delving into Isobe and co-workers’ impressive synthetic work and the intriguing concept from which it was born, it would be instructive to take a few steps back and consider the nuts and bolts of polycyclic aromatic hydrocarbons (PAH) and PAH-based carbon allotropes.

The basic building block of organic chemistry is the carbon atom and it comes in three distinct forms (*sp*^3^, *sp*^2^ and *sp*-hybridized), which differ markedly in their shape and chemical nature. The trigonal planar *sp*^2^-hybridized version of the carbon atom stands out because it is the most closely associated with the concept of aromaticity and it is the only one that gives rise to numerous known allotropes. The assembly of six *sp*^2^-carbon atoms in a cyclic fashion is very favourable from both a geometric and an electronic standpoint because the sigma framework is not distorted in any way and the pi electrons fall into in a very low energy state. Further fusion of six-membered rings gives rise to various series of PAHs and, when done infinitely in two dimensions, to graphene. The inclusion of embedded non-six-membered rings brings with it deviations from planarity and precisely twelve appropriately placed five-membered rings gives rise to the closed three-dimensional carbon allotropes known as the fullerenes. Nonplanar allotropes can also be generated by rolling up sheets of graphene in various ways to give the various types of carbon nanotubes.

Now imagine replacing the *sp*^2^-C building block with a 1,3,5-trisubstituted benzene ring (a “phenine” unit) (Fig. 1). The phenine concept is the brainchild of Prof. Hiroyuki Isobe at the University of Tokyo. It is an open-ended concept because, at first blush, it could conceivably be applied to any *sp*^2^-C-based compound. Isobe and his group have progressed in leaps and bounds from a phenine benzene ([6]cyclo-*meta*-phenylene)^1^, to a phenine corannulene^2^ and then a phenine [7]circulene^3^. The next giant leap was the synthesis of a phenine nanotube (pNT)^4^, which in the *sp*^2^-C world is a (12,12) carbon nanotube segment with regularly positioned holes in it (atom vacancy defects). Isobe et al. have now extended this to the synthesis of a nitrogen-doped version (NpNT) of this large and atomically precise segment of a (12,12) carbon nanotube^5^.

**Fig. 1:**
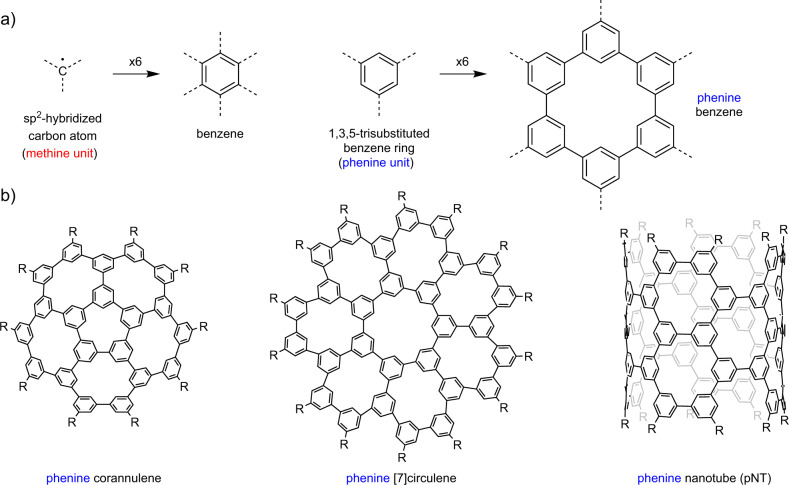
Depiction of the phenine concept and molecules that have been synthesized using it. **a** The essence of the phenine concept; **b** examples of phenine molecules reported by Isobe^2–4^.

Of course, no concept, no matter how clever it is, can be realized without reliable synthetic strategies and robust synthetic methodology behind it. Both of these qualities are strikingly evident in Isobe’s synthesis. A natural consequence of the phenine concept is that most (if not all) C–C bonds formed in the synthesis of a phenine-expanded system are biaryl bonds. The availability of several highly effective transition metal-catalyzed cross- and homocoupling reactions thus provides a solid foundation for the development of general strategies. The synthesis of the (12,12)-NpNT was accomplished using a route that closely resembles the one employed earlier for the synthesis of the analogous all-carbon pNT^4^. This is by no means a case of the same old thing, but rather a testament to the strength of the overarching strategy.

The first stage of the synthesis was the five-step construction of a suitably decorated phenine-expanded benzene (**3**) from commercial 1-bromo-3-chlorobenzene (**1**) and 2,6-dibromopyridine (**2**) (the source of N atoms in the final product) (Fig. 2)^5^. The noteworthy features here are the use of the Marder borylation on two occasions to establish the key 1,3,5 substitution pattern and Suzuki-Miyaura and Yamamoto coupling reactions to stitch together the [6]cyclo-*meta*-phenylene structure. A Yamago-type platinacycle formation then gave rise to oligoarylene **4** after ligand exchange and reductive elimination. The yield for this two-step sequence was just 16%, but this should be weighed against what is accomplished: a giant macrocyclization to give an extraordinarily large oligoarylene (24 aromatic units). The remainder of the skeletal atoms were introduced using a two-step sequence consisting of iododesilylation and another Suzuki-Miyaura cross-coupling to afford **5**. Considering that both transformations involve 16-fold reactions, the 59% yield over two steps is simply amazing (98.4% per reaction). The metamorphosis from nanoring to nanotube was then orchestrated through an 8-fold intramolecular Yamamoto coupling, which delivered the (12,12)-NpNT in 37% yield (88.3% per reaction). Compared to the synthesis of the previously reported pNT^4^, the overall yield improved by a factor of 2 (to 1.4%), which is meaningful in light of the large number of C–C bonds that were formed over the ten-step sequence. The spectacular performance of cross-coupling reactions (Suzuki-Miyaura, Yamago, Yamamoto) certainly bodes well for the construction of larger and even more elaborate systems.

**Fig. 2:**
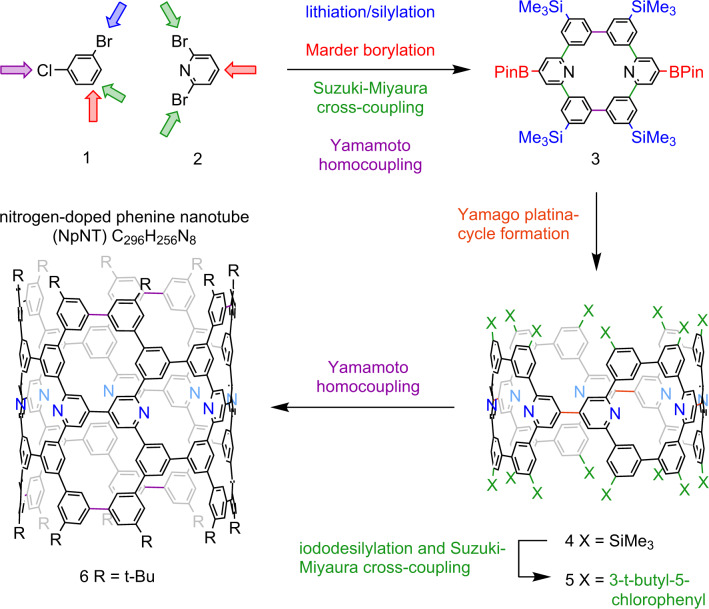
Summary of Isobe and co-workers’ synthesis of a nitrogen-doped phenine nanotube (NpNT). Abbreviated depiction of Isobe and team’s synthesis of a (12,12)-NpNT illustrating key reactions and the sites at which the key carbon-carbon bond-forming reactions occur.

A closer look at the synthesis of the (12,12)-NpNT through the lens of the phenine concept reveals that macrocycle **3** and the (12,12)-NpNT are phenine-expanded versions of compounds that have not yet been synthesized: [4]cyclo-*para*-phenylene and a Vögtle belt corresponding to a slice of a (4,4) carbon nanotube, respectively. Neither of these compounds would be expected to be at all stable and this has a fascinating implication. The phenine concept actually holds much more promise than was apparent at first glance. It has the potential to do more than just recreate what can be done using *sp*^2^-C building blocks. This point is reinforced by the presence of the eight N atoms in the (12,12)-NpNT structure. It is the first demonstration of the obvious, but important point that a phenine unit has the capacity to be modified chemically in a variety of ways, while an *sp*^2^-C atom does not. In addition to the advantages of size, flexibility and the potential for modification, the phenine building block comes with an intact Clar sextet as its π component, whereas an *sp*^2^-C building block comes with an unpaired electron in a *p* orbital (Fig. 3). Consequently, the assembly of *sp*^2^-C atoms can lead both to very favourable and very unfavourable π electronic situations, e.g., as in cyclobutadiene. This limitation is clearly absent when building with phenine units.

**Fig. 3:**
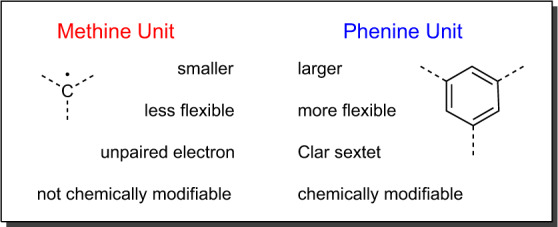
Comparison of the characteristics of the methine and phenine units. Side-by-side listing of the key features of the methine and phenine units, which contribute to their versatility as building blocks for the construction of organic molecules.

Focussing now on the molecule itself, Isobe’s (12,12)-NpNT is a 40-arene behemoth with a molar mass of nearly 4000. It maps onto a (12,12) armchair carbon nanotube and has a length index of *t*_f_ = 7.0. It has exactly eight atom vacancy sites and eight methine units have been replaced with N atoms, quantitative measures of which are the atom-filling and bond-filling indices *F*_a_ (64%) and *F*_b_ (53%), respectively. Despite its great size, the exact structure was fully supported using the standard analytical tools of organic chemistry, including NMR, electronic absorption and fluorescence spectroscopy, mass spectrometry and X-ray crystallography. The latter technique deserves comment. Some very high level crystallography involving charge density methods (specifically, the use of the spherical independent atom model) was used to solve the crystal structure of the nanometer-long molecule, while the use of a multipole transferable aspherical atom model was adopted to achieve an accurate model, which had a minor disorder (12% occupancy) arising from a 45° rotation about the long axis of the cylinder. The exact locations of the nitrogen atoms were pinpointed from the short C–N bond lengths (1.33 Å) and the areas of high electron density corresponding to their lone pairs.

Thus, with complete confidence about the precise atomic structure, Isobe et al. were able to bring a clear set of experimental results to the question of how pyridinic nitrogen-doping influences the properties of single-walled carbon nanotubes. The ability to study a pure molecular substance contrasts what is being learned using top-down methods, where carbon nanotubes, which themselves are complex mixtures, can only be chemically modified in an imprecise fashion. For example, nitrogen-doping can be achieved through exposure of nanotubes to nitrogen plasma, where the achievement of even modest levels of control with respect to regularity and the type of nitrogen-doping (graphitic, pyridinic or pyrrolic) is very difficult to achieve^6^. The main lesson learned from a comparison of the measured and calculated properties of the (12,12)-NpNT to those of the previously reported all-carbon pNT is that pyridinic nitrogen-doping results in a lowering of the energy of the LUMO, but does not affect the HOMO. Based on these findings, nanotubes doped with pyridinic nitrogen atoms can be expected to behave as n-type organic semiconductors.

Isobe’s phenomenal (12,12)-NpNT has provided a solid basis for the development of a more detailed understanding of how pyridinic nitrogen-doping affects the properties of nanotubes and it is easy to imagine that further modifications to the nanotube structure should be achievable through the judicious choice of building blocks and/or synthetic manipulation. A higher number of N atoms could be incorporated, thereby achieving much higher doping levels. The introduction of functional groups into the pores of the phenine nanotube through functionalization of either C or N sites would bring a range of opportunities for function. One could also consider patching the holes through chemical synthesis to give pristine, monodisperse nanotubes, with or without graphitic nitrogen atoms. The synthesis of longer tubes or even those with different roll-up motifs are further attractive possibilities, as is the capping of a tube at one end or both. In the broader picture, the prospect of exploiting the phenine concept to gain access to structure types that are either extremely difficult or downright impossible to construct using *sp*^2^-C atoms is a most exciting one. The possibilities are seemingly infinite.
